# Characterization of Cell Envelope Multiple Mutants of *Brucella ovis* and Assessment in Mice of Their Vaccine Potential

**DOI:** 10.3389/fmicb.2018.02230

**Published:** 2018-09-20

**Authors:** Rebeca Singh Sidhu-Muñoz, Pilar Sancho, Axel Cloeckaert, Michel Stanislas Zygmunt, María Jesús de Miguel, Carmen Tejedor, Nieves Vizcaíno

**Affiliations:** ^1^Departamento de Microbiología y Genética, Universidad de Salamanca, Salamanca, Spain; ^2^Instituto de Investigación Biomédica de Salamanca, Salamanca, Spain; ^3^Plasticité Génomique, Biodiversité, Antibiorésistance (PGBA), UR1282 – Infectiologie Animale, Santé Publique (IASP-311), Institut National de la Recherche Agronomique Centre Val de Loire, Nouzilly, France; ^4^Unidad de Producción y Sanidad Animal, Centro de Investigación y Tecnología Agroalimentaria de Aragón, Instituto Agroalimentario de Aragón – IA2, Zaragoza, Spain

**Keywords:** *Brucella ovis*, outer membrane, virulence, recombinant vaccine, Omp31, Omp25/Omp31 family, cyclic glucans, lipoprotein

## Abstract

*Brucella ovis* is a non-zoonotic *Brucella* species lacking specific vaccine. It presents a narrow host range, a unique biology relative to other *Brucella* species, and important distinct surface properties. To increase our knowledge on its peculiar surface and virulence features, and seeking to develop a specific vaccine, multiple mutants for nine relevant cell-envelope-related genes were investigated. Mutants lacking Omp10 plus Omp19 could not be obtained, suggesting that at least one of these lipoproteins is required for viability. A similar result was obtained for the double deletion of *omp31* and *omp25* that encode two major surface proteins. Conversely, the absence of major Omp25c (proved essential for internalization in HeLa cells) together with Omp25 or Omp31 was tolerated by the bacterium. Although showing important *in vitro* and *in vivo* defects, the Δ*omp10*Δ*omp31*Δ*omp25c* mutant was obtained, demonstrating that *B. ovis* PA survives to the simultaneous absence of Omp10 and four out seven proteins of the Omp25/Omp31 family (i.e., Omp31, Omp25c, Omp25b, and Omp31b, the two latter naturally absent in *B. ovis*). Three multiple mutants were selected for a detailed analysis of virulence in the mouse model. The Δ*omp31*Δ*cgs* and Δ*omp10*Δ*omp31*Δ*omp25c* mutants were highly attenuated when inoculated at 10^6^ colony forming units/mouse but they established a persistent infection when the infection dose was increased 100-fold. The Δ*omp10*Δ*ugpB*Δ*omp31* mutant showed a similar behavior until week 3 post-infection but was then totally cleared from spleen. Accordingly, it was retained as vaccine candidate for mice protection assays. When compared to classical *B. melitensis* Rev1 heterologous vaccine, the triple mutant induced limited splenomegaly, a significantly higher antibody response against whole *B. ovis* PA cells, an equivalent memory cellular response and, according to spleen colonization measurements, better protection against a challenge with virulent *B. ovis* PA. Therefore, it would be a good candidate to be evaluated in the natural host as a specific vaccine against *B. ovis* that would avoid the drawbacks of *B. melitensis* Rev1. In addition, the lack in this attenuated strain of Omp31, recognized as a highly immunogenic protein during *B. ovis* infection, would favor the differentiation between infected and vaccinated animals using Omp31 as diagnostic target.

## Introduction

*Brucella ovis* is a Gram-negative bacterial species belonging to the genus *Brucella*. It is a non-zoonotic species mainly provoking epididymitis and other genital lesions in rams, although it has also been associated with increased perinatal mortality in lambs and placentitis, abortions, and infertility in ewes (OIE, 2017b). It causes significant economic losses worldwide and lacks commercially available specific vaccine. *B. ovis* lipopolysaccharide (LPS) is devoid of *O*-polysaccharide (O-PS) chains and is defined as rough (R) LPS (R-LPS). *B. ovis* and *B. canis* are the sole species of the *Brucella* genus constituted exclusively by R strains that are virulent for their natural hosts. This characteristic differentiates them from smooth (S) brucellae that require O-PS for full virulence (e.g., *B. melitensis, B. abortus*, or *B. suis*). *B. melitensis* Rev1, currently used for vaccination against ovine and caprine brucellosis caused by *B. melitensis*, is considered the best available vaccine against *B. ovis* (OIE, 2017a). However, this vaccine is banned in countries or regions where infection by *B. melitensis* is eradicated because, among other drawbacks, it induces antibodies that interfere with the serological diagnosis of infections caused by S brucellae. Therefore, the development of a specific vaccine for the prophylaxis of *B. ovis* infection is a matter of interest. Considering that the best available vaccines against brucellosis caused by S *Brucella* strains are homologous S attenuated strains (Nicoletti, 2010), the search for a *B. ovis* attenuated vaccine strain seems an interesting approach. The first step to achieve this goal is the identification of virulence factors that can be removed from *B. ovis*, to minimize its deleterious effects on the host, but without compromising its immunogenicity.

Comparatively to its S counterparts, little is known about the virulence of *B. ovis*, although a species-specific ABC transporter (Silva et al., 2011, 2013) and some classical virulence factors described in S species have been identified as necessary for its virulence (Martín-Martín et al., 2012; Sá et al., 2012; Macedo et al., 2015). O-PS chains mask other outer membrane (OM) components in S strains (Cloeckaert et al., 2002), hindering their interaction with host cells, antibodies, and other elements of the immune system. According to the surface exposure of OM molecules other than LPS in R *B. ovis*, a more important role in the interaction with host cells and virulence than in S strains would be expected. However, several OM proteins (OMPs) and OM-related genes necessary for full virulence in S strains seem not required in *B. ovis* experimental infection models (Martín-Martín et al., 2012; Sidhu-Muñoz et al., 2016). This observation reveals differences among the brucellae regarding the role of the OM molecules in host–pathogen interactions, differences that might be associated with their heterogeneity regarding OM-related properties (Martín-Martín et al., 2011; Vizcaíno and Cloeckaert, 2012), host-preference, and pathogenicity. Although the *Brucella* species share a high level of DNA homology, an increased number of pseudogenes and insertion sequences has been detected in *B. ovis*, when compared to zoonotic S *Brucella* (Tsolis et al., 2009). This feature led to hypothesize that its narrow host-range and tissue tropism (almost exclusively restricted to ovine male genital tract) is in part consequence of genome degradation (Tsolis et al., 2009). However, despite this genome degradation, that among others affects O-PS biosynthetic genes and several OMPs (Tsolis et al., 2009), *B. ovis* causes a chronic infection in its natural host and in laboratory animals (Caro-Hernández et al., 2007; Silva et al., 2011; OIE, 2017b), which would also support a specific pattern of interaction between the host and the bacterial OM.

With the aim of increasing our knowledge about the contribution of cell envelope components to OM-related properties and virulence of *B. ovis* and as a tool to develop a specific live attenuated vaccine, in this work we have constructed and characterized a panel of *B. ovis* multiple mutants in genes related to the cell envelope that either code for major OMPs or either are individually required in S *Brucella* strains, but not in *B. ovis*, for virulence (Caro-Hernández et al., 2007; Martín-Martín et al., 2012; Sidhu-Muñoz et al., 2016). Genes targeted in multiple mutations were: (i) *omp31, omp25*, and *omp25c* that code for major OMPs in *B. ovis* (Cloeckaert et al., 2002; Martín-Martín et al., 2009); (ii) *omp10* and *omp19* that encode two minor OM lipoproteins (Tibor et al., 1999) required in *B. abortus* 544 for full virulence (Tibor et al., 2002); (iii) *bepC* that encodes a TolC-homolog protein necessary in *B. suis* 1330 for full virulence (Posadas et al., 2007); (iv) *bacA* that encodes an integral inner membrane protein involved in lipid A acylation, cell-envelope properties, and virulence in *B. abortus* 2308 (LeVier et al., 2000; Roop et al., 2002; Ferguson et al., 2004; Parent et al., 2007); and (v) *ugpB* that encodes SP41, a surface protein involved in invasion of *B. suis* 1330 to HeLa cells (Castañeda-Roldán et al., 2006). Beside these genes that are not individually required for virulence in *B. ovis* PA, multiple mutations also included *cgs*, involved in the synthesis of periplasmic cyclic β-1,2-glucans (CβGs) (Iñón de Iannino et al., 1998; Haag et al., 2010) and necessary for full virulence in *B*. *abortus* 2308 (Briones et al., 2001). The Δ*cgs* mutant of *B. ovis* PA was also highly attenuated when it was intraperitoneally inoculated at a dose of 10^6^ colony forming units (CFU)/mouse (Martín-Martín et al., 2012), but when the dose usually employed for protection experiments (10^8^ CFU/mouse) (Sancho et al., 2014; Soler-Lloréns et al., 2014; Silva et al., 2015b) was used, the bacterial counts in spleen increased to levels that were close to those of the parental strain (unpublished results). Several combinations of deleted genes were tempted in the panel of multiple mutants, although, keeping in mind the development of an attenuated vaccine, the inclusion of Δ*cgs* and Δ*omp31* mutations was prioritized for two reasons: (i) to study how the addition of new mutations could increase the attenuation of the Δ*cgs* single mutant to appropriate levels for an attenuated vaccine, and (ii) Omp31 is an abundant OMP in the OM of *B. ovis* PA (Martín-Martín et al., 2009), it is the most immunogenic OMP in the course of *B. ovis* infection (Kittelberger et al., 1998) and its interest for a serological diagnosis favoring the differentiation between infected and vaccinated animals (DIVA diagnosis) has also been evidenced (Vizcaíno et al., 2001b).

## Materials and Methods

### Bacterial Strains, Culture Conditions, and Plasmids

*Brucella ovis* PA and *B. melitensis* Rev1 were obtained from BCCN (*Brucella* Culture Collection Nouzilly, maintained at the Institut National de la Recherche Agronomique, Nouzilly, France), and the other *B. ovis* strains are listed in **Table 1**. Recombinant plasmids used for mutagenesis of *cgs* (BOV_RS00 535), *bacA* (BOV_RS01960), *omp10* (BOV_RS10700), *omp19* (BOV_RS09115), *bepC* (BOV_RS04655), and *ugpB* (BOV_RS13470), and the corresponding single mutants derived from parental *B. ovis* PA have previously been described (Martín-Martín et al., 2012; Sidhu-Muñoz et al., 2016). *B. ovis* Δ*omp31* (BOV_RS12205) and Δ*omp25* (BOV_RS3460) single nonpolar mutants (**Table 1**) were obtained from parental *B. ovis* PA as described below. Multiple mutants (**Table 1**) were constructed from initial single mutants where one or two of the mentioned genes were additionally deleted. The Δ*omp31*-k and Δ*omp25c*-k mutant strains (*omp31* or *omp25c* replaced by a kanamycin resistance cassette) and the same mutants complemented with the corresponding *omp31* or *omp25c* (BOV_RS00575) wild type genes (Δ*omp31*-k com and Δ*omp25c*-k com strains) were previously obtained (Caro-Hernández et al., 2007).

**Table 1:** Most relevant bacterial strains used in this work, growth characteristics, and preliminary evaluation of virulence.

			Log CFU/spleen at week (W) p.i.^c^			
	Deleted gene/s and strain	Log CFU/ml	(dose 10^6^ CFU)		(dose 10^8^ CFU)	
*Brucella ovis* strains^a^	abbreviation in the text	OD_600_ = 0.2^b^	W3	W7	W3	W7
*B. ovis* PA (BCCN 76-250)	Parental strain, PA	9.09 0.04	6.90	5.85	7.62	6.31
**Single mutants**						
*B. ovis*-pPS31OVL02M	Δ*omp31*	8.80 0.10*	5.44	5.79	–	–
*B. ovis*-pNV25OVL02M	Δ*omp25*	9.08 0.07	6.87	4.92	–	–
*B. ovis*-PNV25cA	Δ*omp25c-k*	8.93 0.03*	7.20	5.61	–	–
*B. ovis*-pNVcgs03M	Δ*cgs*	8.88 0.08*	0.45	0.53	6.20	5.53
*B. ovis*-pNVbacA03M	Δ*bacA*	9.12 0.07	6.48	5.64	–	–
*B. ovis*-pNV1002M	Δ*omp10*	8.84 0.04*	6.91	5.79	–	–
*B. ovis*-pNV1902M	Δ*omp19*	8.92 0.05*	6.51	5.90	–	–
*B. ovis*-pNVBepC02M	Δ*bepC*	9.15 0.08	6.17	5.28	–	–
*B. ovis*-pNVSP4102M	Δ*ugpB*	9.10 0.03	7.72	6.30	–	–
**Double mutants**						
*B. ovis* Δ*omp31*-pNV1902M	Δ*omp31*Δ*omp19*	8.78 0.04*	5.25	5.92	–	–
*B. ovis* Δ*omp31*-pNVBepC02M	Δ*omp31*Δ*bepC*	8.80 0.04*	6.78	5.75	–	–
*B. ovis* Δ*omp31*-pNVSP4102M	Δ*omp31*Δ*ugpB*	8.82 0.06*	5.90	6.32	–	–
*B. ovis* Δ*omp31*-pNVcgs03M	Δ*omp31*Δ*cgs*	8.49 0.09*	–	–	5.16	4.95
*B. ovis* Δ*omp25*-pNVcgs03M	Δ*omp25*Δ*cgs*	8.56 0.06*	–	–	5.52	5.15
*B. ovis* Δ*omp25*-pNV25cOVL02M	Δ*omp25*Δ*omp25c*	8.82 0.06*	6.91	6.32	–	–
*B. ovis* Δ*cgs*-pNVSP4102M	Δ*cgs*Δ*ugpB*	8.89 0.04*	–	–	5.24	4.80
*B. ovis* Δ*cgs*-pNVBepC02M	Δ*cgs*Δ*bepC*	8.87 0.07*	–	–	5.45	4.63
*B. ovis* Δ*omp10*-pPS31OVL02M	Δ*omp10*Δ*omp31*	8.89 0.07*	6.72	6.66	–	–
*B. ovis* Δ*omp10*-pNVSP4102M	Δ*omp10*Δ*ugpB*	8.91 0.06*	7.29	5.66	–	–
*B. ovis* Δ*omp10*-pNVcgs03M	Δ*omp10*Δ*cgs*	8.82 0.09*	–	–	5.21	5.39
*B. ovis* Δ*omp19*-pNVcgs03M	Δ*omp19*Δ*cgs*	8.79 0.03*	–	–	6.07	5.99
*B. ovis* Δ*omp19*-pNVSP4102M	Δ*omp19*Δ*ugpB*	8.88 0.03*	6.57	5.36	–	–
*B. ovis* Δ*bepC*-pNVSP4102M	Δ*bepC*Δ*ugpB*	9.10 0.05	7.37	5.63	–	–
*B. ovis* Δ*bacA*-pPS31OVL02M	Δ*bacA*Δ*omp31*	8.81 0.02*	6.05	5.36	–	–
**Triple mutants**						
*B. ovis* Δ*omp10*Δ*ugpB-*pPS31OVL02M	Δ*omp10*Δ*ugpB*Δ*omp31*	8.76 0.18*	0.47	0.57	5.53	0.52
*B. ovis* Δ*omp10*Δ*omp31-*pNV25cOVL02M	Δ*omp10*Δ*omp31*Δ*omp25c*	8.49 0.04*	2.57	3.26	5.30	5.70
*B. ovis* Δ*omp31*Δ*omp10-*pNV25cOVL02M	Δ*omp31*Δ*omp10*Δ*omp25c*	8.39 0.03*	2.42	2.03	–	–
*B. ovis* Δ*omp31*Δ*cgs-*pNV1002M	Δ*omp31*Δ*cgs*Δ*omp10*	8.61 0.07*	–	–	5.53	5.73
*B. ovis* Δ*omp31*Δ*cgs-*pNV1902M	Δ*omp31*Δ*cgs*Δ*omp19*	8.71 0.05*	–	–	5.07	4.61
*B. ovis* Δ*omp31*Δ*bepC-*pNVSP4102M	Δ*omp31*Δ*bepC*Δ*ugpB*	8.86 0.12*	7.65	6.00	–	–
**Other previous mutants used as controls**						
*B. ovis* PNV31A	Δ*omp31*-k	8.85 0.03*	–	–	–	–
*B. ovis* PNV31A-com	Δ*omp31*-k com	8.89 0.03*	–	–	–	–
*B. ovis* PNV25c-com	Δ*omp25c*-k com	8.99 0.06	–	–	–	–

*^*a*^Log CFU/spleen 3 and 7 weeks after intraperitoneal infection with 1 × 10^*6*^ or 1 × 10^*8*^ CFU/mouse. One mouse was used per time point in this preliminary assay of virulence. –, not determined. ^*b*^The *B. ovis* mutants derive from *B. ovis* PA, which was obtained from BCCN (*Brucella* Culture Collection Nouzilly, Institut National de la Recherche Agronomique, Nouzilly, France). *B. ovis* Δ*cgs*, Δ*bacA*, Δ*omp10*, Δ*omp19*, Δ*bepC*, and Δ*ugpB* were previously described (Martín-Martín et al., 2012; Sidhu-Muñoz et al., 2016). *B. ovis* PA Δ*omp25c*-k and Δ*omp31*-k were also previously obtained replacing *omp25c* and *omp31*, respectively, by a kanamycin resistance cassette; they were complemented with wild-type *omp25c* and *omp31*, respectively, to give *B. ovis* Δ*omp25c*-k com and *B. ovis* Δ*omp31*-k com (Caro-Hernández et al., 2007). The other *B. ovis* mutants were obtained in this work. ^*c*^The asterisk (^∗^) indicates statistically significant differences (*P* < 0.05), compared to the parental strain. Log CFU/ml of bacterial suspensions adjusted to an OD_*600*_ value of 0.2. The results are expressed as the mean ± SD of three assays.*

*Brucella ovis* strains and the *B. melitensis* Rev1 attenuated vaccine were cultured in tryptic soy agar or tryptic soy broth (Pronadisa-Laboratorios Conda, Torrejón de Ardoz, Spain) both supplemented with 0.3% yeast extract (Pronadisa-Laboratorios Conda, Torrejón de Ardoz, Spain) and 5% horse serum (Gibco-Life Technologies, Grand Island, NY, United States) (TSA-YE-HS and TSB-YE-HS, respectively). Incubations were performed at 37°C in a 5% CO_2_ atmosphere and, in the case of TSB-YE-HS liquid medium, under agitation at 120 rpm. When required, streptomycin (Strep; 50 μg/ml) (Sigma-Aldrich, St. Louis, MO, United States) was added to the culture medium of *B. melitensis* Rev1 (Strep-resistant strain). Similarly, when necessary for the selection of the recombinant *B. ovis* strains, kanamycin (50 μg/ml) or 5% sucrose (Sigma-Aldrich, St. Louis, MO, United States) was added. Assays with the *B. ovis* strains, including Δ*omp31*-k, Δ*omp25c*-k, Δ*omp31*-k com, and Δ*omp25c*-k com strains, were performed in the absence of antibiotics.

Plasmid pGEM-T Easy (Promega, Madison, WI, United States) was used to clone PCR-amplified fragments and pCVD-KanD (Martín-Martín et al., 2012) was the suicide plasmid employed to insert the mutant genes into parental *B. ovis* PA. They were propagated in *Escherichia coli* JM109 or CC118 (λpir), respectively, that were incubated at 37°C in Luria Bertani medium supplemented, when required, with ampicillin or kanamycin (50 μg/ml). Their derived recombinant plasmids constructed during this work are mentioned below.

### DNA Primers and Mutagenesis

DNA primers (IDT, Leuven, Belgium) used for the construction and verification of the *B. ovis* Δ*omp31*, Δ*omp25*, and Δ*omp25c* single and multiple nonpolar mutants are listed in **Table 2**. The additional primers used to check the multiple mutants were previously described (Martín-Martín et al., 2012; Sidhu-Muñoz et al., 2016).

**Table 2:** Primers used in this work for the construction and verification of *omp25, omp25c*, and *omp31* single and multiple mutants^a^.

Primer name	Nucleotide sequence 5′–3^′b^	Target gene or plasmid^c^
**Construction of *B. ovis* PA mutants**		
25MUTZ-F	CGACCTTATCCTCCTGAA	*omp25*
25OVL-R	GACGATTACGAGAGACTT	*omp25*
25OVL-F	AAGTCTCTCGTAATCGTCAAGCTGGACACGCAGGAT	*omp25*
25MUTZ-R	TTTGCGACGTTTTGCTGG	*omp25*
25cdMUT-F	TGCGTGGTTCAGATTTCG	*omp25c*
25cOVL-R	AGCCTTGAGCTTCATGAT	*omp25c*
25cOVL-F	ATCATGAAGCTCAAGGCTGCTTACAAGTTCTGATAG	*omp25c*
25cMUT-R	AGCCGTAACCAACCTGAC	*omp25c*
31MUT-F	AGAATAAAACACATGCCC	*omp31*
31OVL-R	GATGGACGCCAAAATTAC	*omp31*
31OVL-F	GTAATTTTGGCGTCCATCGTCGGTCTGAACTACAAG	*omp31*
31MUT-R	GCTGAATGCGGAGATGGT	*omp31*
**Additional primers for the verification of recombinant plasmids**		
**and mutants**		
Universal-F	GTTTTCCCAGTCACGAC	pGEM-T Easy
Universal-R	CAGGAAACAGCTATGAC	pGEM-T Easy
25-Sec	GGACCGCGCAAAACGTAATT	*omp25*
25-MAT	GCCGACGCCATCCAGGAA	*omp25*
25c-MAT	GCTGACGCCGTCATTGAA	*omp25c*
31-MAT	GCCGACGTGGTTGTTTCT	*omp31*

*^*a*^Primers for the verification of proper deletion of other genes have been previously described (Martín-Martín et al., 2012; Sidhu-Muñoz et al., 2016). ^*b*^Underlined sequences in 25OVL-F, 25cOVL-F, and 31OVL-F correspond to regions overlapping with 25OVL-R, 25cOVL-R, and 31OVL-R, respectively. ^*c*^Target gene is the *B. ovis* gene to be deleted or PCR-amplified for the verification of mutant strains. Primers Universal-F and Universal-R target pGEM-T Easy and its derived recombinant plasmids at both sides of the cloned insert and were used for sequencing of the DNA insert. The remaining primers target the *B. ovis* genome and were designed according to the published genome sequence of *B. ovis* 63/290 (ATCC 25840) (accession numbers NC_009505 and NC_009504 for chromosome I and II, respectively).*

For the construction of the recombinant plasmids used in the mutagenesis process, inactivation of *omp31, omp25*, and *omp25c* was performed by in-frame deletion with overlapping PCR (Martín-Martín et al., 2012). Briefly, the 5′- and 3′-ends of each target gene, together with about 300–700 pb upstream or downstream, respectively, were separately amplified by PCR with two pairs of primers (31MUT-F + 31OVL-R and 31OVL-F + 31MUT R for *omp31*, 25MUTZ-F + 25OVL-R and 25OVL-F + 25MUTZ-R for *omp25*, 25cdMUT-F + 25cOVL-R and 25cOVL-F + 25cMUT-R) (**Table 2**). The two amplified fragments were fused, through the overlapping sequences located in the internal primers (primers OVL-F and OVL-R) (**Table 2**), in a PCR reaction with the two external primers of each fragment (31MUT-F + 31MUT-R for *omp31*, 25MUTZ-F + 25MUTZ-R for *omp25*, and 25cdMUT-F + 25cMUT-R for *omp25c*). The resulting mutant genes were cloned in pGEM-T Easy, verified by DNA sequencing, and subsequently cloned in pCVD-KanD to give pPS31OVL02, pNV25OVL02, and pNV25cOVL02, respectively. Plasmids were introduced in *B. ovis* PA by electroporation and the selection of bacteria with the corresponding plasmid integrated in the chromosome (intermediate strains), through a single homologous recombination event, was performed on TSA-YE-HS plates with kanamycin. The selected intermediate strains were plated onto TSA-YE-HS supplemented with 5% sucrose to give either the desired mutant strain (wild-type gene replaced by the inactivated gene) or a strain reverting to the parental genotype (Rv). They were differentiated by PCR amplification with the two primers external to each side of the deleted gene (amplified fragment with higher size in Rv strains than in mutant strains) and a second PCR with an external primer and a primer annealing inside the deleted fragment (primers 31-MAT, 25-MAT, and 25c-MAT) (**Table 2**). The latter PCR reaction produces no amplification in mutant strains.

For multiple gene mutagenesis, single mutants listed in **Table 1** were subjected to a second mutagenesis round with a recombinant plasmid containing another inactivated gene. A third round of mutagenesis was conducted on some selected double mutants to inactivate a third gene. The selection of mutant strains was performed with specific PCRs targeting each inactivated locus.

### Growth Pattern, Autoagglutination, and Susceptibility Assays

Growth characteristics of the mutant strains in TSA-YE-HS plates and TSB-YE-HS liquid medium were compared to those of parental *B. ovis* PA. Numbers of CFU/ml corresponding to bacterial suspensions in PBS with optical density scores at 600 nm (OD_600_) of 0.2 were determined for each mutant by triplicate plating on TSA-YE-HS of the properly diluted suspensions. The initial suspensions were prepared from bacteria cultured in TSA-YE-HS plates for 44 h. Colony size was photographed 5 days after plating and colonies enumerated 8 days after plating. Growth curves were established for triplicate bacterial suspensions in TSB-YE-HS medium (30 ml) with initial OD_600_ readings of 0.05 that were incubated at 37°C under agitation (120 rpm) and a 5% CO_2_ atmosphere. OD_600_ scores were measured through a 120-h period, and CFU/ml numbers were evaluated at the beginning of the experiment (t0), and after 24, 52, and 77 h of incubation (t24, t52, and t77, respectively), by plating the properly diluted cultures on TSA-YE-HS.

The autoagglutination assay was performed as described previously (Caro-Hernández et al., 2007; Martín-Martín et al., 2012) by measuring the evolution, over 48 h of static incubation, of the OD_600_ values of bacterial suspensions with initial readings of 0.8 (100% OD_600_) in TSB-YE-HS. Susceptibility to 1 mg/ml of polymyxin B (Sigma-Aldrich, St. Louis, MO, United States) and 0.1 mg/ml of sodium deoxycholate (Sigma-Aldrich, St. Louis, MO, United States) in PBS was expressed as the percentage of survival after 80 min of exposure. It was determined by comparison of the numbers of CFU in untreated (incubation in PBS, 100% survival) and treated bacterial suspensions (Caro-Hernández et al., 2007; Martín-Martín et al., 2011). The results were expressed as means ± standard deviation (SD) of three assays.

### Mapping of Cell Envelope Antigens

Reactivity of *B. ovis* mutants with MAbs specific for cell-envelope antigens (**Table 3**) was measured by indirect enzyme-linked immunosorbent assay (iELISA) as previously described (Soler-Lloréns et al., 2014). MAbs specific for *Brucella* peptidoglycan (PG), R-LPS, S-LPS, Omp2b, Omp10, Omp16, Omp19, Omp25, Omp31, or periplasmic BP26 were used (Cloeckaert et al., 1990, 1991, 1996a,b; Vizcaíno et al., 2001b; Seco-Mediavilla et al., 2003). Briefly, 96-well plates were coated overnight at room temperature with sonicated (mild homogenization for 10 s at 40% intensity in a Sonic Dismembrator model 120, Thermo Fisher Scientific, Waltham, Ma, United States) bacterial suspensions in PBS (OD_600_ = 1), which were prepared from cultures in TSA-YE-HS plates. MAbs (hybridoma supernatant) diluted 1/2 and a goat anti-mouse IgG-horseradish peroxidase conjugate (Bio-Rad, Hercules, CA, United States) diluted 1:9000 were used as primary and secondary antibodies, respectively. Antigen–antibody binding was revealed by incubation for 20 min with TMB as substrate for peroxidase and subsequent addition of a 1 M HCl-based stop solution (Interchim, Montluçon, France). The results were expressed as means ± SD of the values recorded at 450 nm (OD_450_) in a Labsystems Multiskan Ascent microplate reader (Thermo Fisher Scientific) for three repeats by MAb and strain.

**Table 3:** Main characteristics of the monoclonal antibodies used in this work.

Monoclonal antibody^a^	Specificity	Abbreviation
A68/15B06/C08	Omp2b	C08
A68/07G11/C10	Omp10	C10
A76/08C03/G03	Omp16	G03
A76/18B02/D06	Omp19	D06
A59/10F09/G10	Omp31	G10
A59/05F01/C09	Omp25	C09
A18/13D02/F05	Omp25	F05
A76/08H09/A02	Omp25	A02
V78/09B12/B02	BP26	B02
V78/02E08/F03	BP26	F03
V78/04D01/A10	BP26	A10
V78B/04G07/H05	BP26	H05
A76/03D06/A09	PG	A09
A76/12G12/F12	S-LPS	F12
A68/03F03/D05	R-LPS	D05

*^*a*^The MAbs were obtained and characterized previously (Cloeckaert et al., 1990, 1991, 1992, 1996a,b; Zygmunt et al., 1994; Seco-Mediavilla et al., 2003).*

Additionally, sodium dodecyl sulfate polyacrylamide gel electrophoresis (SDS–PAGE) and immunoblot were also performed and carried out as previously described (Vizcaíno et al., 2001b; Martín-Martín et al., 2009). Briefly, bacterial suspensions concentrated to OD_600_ values of 20 were prepared in H_2_O with the different *B. ovis* strains. They were submitted to SDS–PAGE on a Protean II xi cell (Bio-Rad, Hercules, CA, United States) and either stained with Coomassie blue (Bio-Rad, Hercules, CA, United States) or transferred to a nitrocellulose membrane in a semidry electroblotter (Amersham, GE Healthcare, Little Chalfont, United Kingdom). Prestained protein marker VI (Applichem-Panreac, Barcelona, Spain) was used as protein standard. Nitrocellulose strips were saturated with skim milk and then incubated with sera obtained, as described before (Martín-Martín et al., 2009), by immunization of rabbits with Omp31b, Omp25c, Omp25d, and Omp22 purified recombinant proteins. Binding of the secondary antibody – a goat anti-rabbit IgG-peroxidase conjugate (Sigma-Aldrich, St. Louis, MO, United States) – was detected with a 4-chloro-1-naphthol substrate solution.

### Infection Assays on Murine Macrophage and HeLa Cells

Infection assays of murine macrophage-like J774.A1 cells (DSMZ ACC170) and epithelial HeLa (ATCC CCL-2TM) cells were performed as described previously (Sidhu-Muñoz et al., 2016). Briefly, 2 × 10^4^ J774.A1 macrophages or 1.5 × 10^4^ HeLa cells/well were seeded on 96-well sterile microplates and incubated for 24 h at 37°C under a 5% CO_2_ atmosphere. After incubation for 2 h with the *B. ovis* strains (4 × 10^6^ CFU/well for macrophages or 8 × 10^6^ CFU/well for HeLa cells) and killing of extracellular bacteria by incubation with gentamycin for 1 h, intracellular bacteria were enumerated in three wells per strain after lysis of the phagocytes with H_2_O [t0 post-infection (p.i.)] (Sidhu-Muñoz et al., 2016). The remaining wells were maintained in the presence of gentamycin and intracellular bacteria were similarly determined at 20 (t20) and 44 h (t44) p.i. The results were expressed as means ± SD of the log CFU/well at each selected p.i. time point (t0, t20, and t44) and are representative of at least two experiments.

### Mice and Ethics Statement

Female 6-week-old BALB/c mice (Charles River Laboratories, Chatillon-sur-Chalaronne, France), received 1 week previously, were used. They were randomly distributed into experimental groups and kept with water and food *ad libitum* in the animal experimentation facilities of the University of Salamanca (registration number PAE SA-001) or the Unidad de Producción y Sanidad Animal, Instituto Agroalimentario de Aragón-IA2 (CITA-Universidad de Zaragoza) (registration number ES502970012005).

Procedures with mice were designed according to Spanish and European legislation regarding the use of animals in research (RD 53/2013 and directive 2010/63/UE). Microbiological practices and animal experimentation were approved by the Biosecurity and Bioethics Committees of the University of Salamanca, and authorized by the competent authority of Junta de Castilla y León, Spain. The Animal Welfare Committee of the CITA (Spain) also reviewed and approved the protocols.

### Virulence Assays and Antibody Response in Mice

Preliminary evaluation of virulence in mice was performed by intraperitoneal inoculation of 10^6^ or 10^8^ CFU/mouse depending on the previous information about each single mutant (Martín-Martín et al., 2012; Sidhu-Muñoz et al., 2016). One mouse was used per strain, dose and time of analysis and splenic colonization was evaluated 3 or 7 weeks (W3 and W7) p.i. as described previously (Sancho et al., 2014). These time points in parental *B. ovis* PA correspond to the acute and chronic phase of infection, respectively (Caro-Hernández et al., 2007; Martín-Martín et al., 2012; Sidhu-Muñoz et al., 2016).

Spleen colonization of the selected mutants – according to the results of the preliminary analysis – was evaluated at W3 and W7 p.i. in mice inoculated intraperitoneally with 10^6^ CFU, and at W1, W3, W5, W7, and W11 p.i. in mice inoculated intraperitoneally with 10^8^ CFU. Five mice per strain and time point were used. The results were expressed as means ± SD (*n* = 5) of the log of CFU/spleen for each strain and time point. The identity of the recovered colonies was checked by PCR. Antibodies specific for *B. ovis* PA were determined by iELISA (Sancho et al., 2014) in sera obtained from submandibular blood from the same mice. Briefly, a suspension in PBS of heat-inactivated *B. ovis* PA whole cells (OD_600_ = 1) was used as the coating antigen of 96-well plates and a goat antimouse IgG-peroxidase conjugate (Sigma–Aldrich, St. Louis, MO, United States) was used as the secondary antibody. OD_405_ readings were recorded on a Multiskan Go Microplate Reader (Thermo Fisher Scientific) after 30 min incubation at room temperature with the substrate solution constituted by 1 mM 2,2′-azino-di-(3-3-ethylbenzothiazoline-sulfonic acid) (ABTS; Sigma-Aldrich, St. Louis, MO, United States) and 2 mM H_2_O_2_ (Sigma-Aldrich, St. Louis, MO, United States) in 0.1 M citrate, pH 4.2. Antibody titers in serum were defined as the inverse of the highest serum dilution scoring an OD_405_ value twice as high as that obtained with the blank (mean OD_405_ of six wells in which serum was replaced by dilution buffer). The results were represented as means ± SD of the log of the titers obtained with five mice analyzed individually.

### Vaccine Efficacy and Immune Response of *B. ovis* Δ*omp10*Δ*ugpB*Δ*omp31*

BALB/c mice were inoculated intraperitoneally with PBS, 10^5^ CFU of the classical vaccine *B. melitensis* Rev1, or 10^8^ CFU of the attenuated mutant *B. ovis* Δ*omp10*Δ*ugpB*Δ*omp31.* Seven weeks later, corresponding with the time point where the *B. ovis* vaccine was cleared from spleen, five mice per group were either challenged with 2 × 10^5^ CFU of *B. ovis* PA, or processed for the evaluation of the antibody and cellular immune responses specific for *B. ovis* PA. Determination of virulent *B. ovis* PA in spleen was evaluated 3 weeks after experimental challenge (Sancho et al., 2014). The CFU number of virulent *B. ovis* PA in mice vaccinated with *B. melitensis* Rev 1 was obtained by subtracting the values obtained in TSA-YE-HS-Strep medium from those obtained in the same medium without antibiotic. Results were expressed as means ± SD (*n* = 5) of the log CFU/spleen of *B. ovis* PA for each vaccination group.

IgG titers in serum were analyzed, as described above, in five mice per group 7 weeks after vaccination by using heat-inactivated *B. ovis* PA whole cells as the coating antigen in a iELISA test. Additionally, IgG isotypes were determined under the same conditions but using goat anti-mouse IgG_1_-, IgG_2a_-, or IgG_2b_-peroxidase conjugates (Santa Cruz Biotechnology, Dallas, TX, United States). The same mice were processed as previously described (Sancho et al., 2014) to evaluate the cytokine response of splenocytes to a second stimulus with *B. ovis* PA. Briefly, spleen cells from immunized mice were cultured in 24-well sterile plates and stimulated by exposure to heat-inactivated (1 h at 65°C) *B. ovis* PA whole cells (10^7^ CFU/well), 10 μg/ml of the mitogen concanavalin A (Sigma-Aldrich, St. Louis, MO, United States) as a positive control of cell proliferation, or culture medium as a negative control. After 72 h of incubation, the culture supernatants were harvested to evaluate the levels of interferon-γ (IFN-γ), tumor necrosis factor-α (TNF-α), IL-10, and IL-12(p40). They were determined by sandwich ELISA with OptEIA^TM^ Mouse Sets specific to each cytokine, as instructed by the manufacturer (BD Biosciences, San Diego, CA, United States). Two wells were used for each experimental condition and mouse. The results for each vaccination group were expressed as means ± SD of the cytokine amount (ng/well) in the supernatants of splenocytes obtained from five individual mice. The results obtained with the positive and negative controls (concanavalin A and RPMI as stimulating agents, respectively) were as expected and are not shown.

### Statistical Analysis

Statistical comparisons between means were performed using analysis of variance. The significance of the differences (*P* < 0.05) between the experimental groups was determined with the *post hoc* Fisher’s protected least significant differences (PLSD) test. To simplify the figures and tables, no ranking of *P-*values has been established and all significant differences are marked as *P* < 0.05.

## Results

### Genotypic Characterization of the Mutants

The *B. ovis* mutants (**Table 1**) were genotypically characterized as described in the section “Materials and Methods.” Two PCR reactions were settled for each inactivated gene, one reaction with external primers to each side of the deleted locus (amplified fragment of lower size in mutant strains than in parental strain), and a second reaction with an external primer to the deleted loci and a primer annealing inside the deleted region (no amplification in mutant strains). All single and multiple mutants gave the expected results for each inactivated gene (data not shown).

Although not all combinations of single genes shown in **Table 1** were tempted in multiple mutants, two combinations were specially assayed without success: a double mutant in lipoproteins Omp10 and Omp19 and a double mutant in major Omp25 and Omp31 proteins. Despite multiple attempts, the intermediate strains for these double mutants always reverted to the single mutant initial genotype. Similarly, two independent *omp25c* intermediate strains always reverted to the parental genotype and, consequently, the non-polar Δ*omp25c* single mutant was not obtained. However, the non-polar deletion of *omp25c*, with plasmid pNV25cOVL02, was successful in the Δ*omp25*Δ*omp25c* double mutant and in the Δ*omp10*Δ*omp31*Δ*omp25c* and Δ*omp31*Δ*omp10*Δ*omp25c* triple mutants. The Δ*omp25c*-k mutant, where *omp25c* was replaced by a kanamycin resistance cassette (Caro-Hernández et al., 2007), was used in this work for comparisons with the non-polar Δ*omp25c* double and triple mutants.

### Growth Characteristics of the Mutants

The numbers of CFU in bacterial suspensions with OD_600_ values of 0.2 were determined for each mutant in TSA-YE-HS plates. Bacterial counts obtained for Δ*omp25*, Δ*bepC*, Δ*ugpB*, and Δ*bacA* single mutants and for Δ*bepC*Δ*ugpB* double mutant did not significantly differ from those observed with the parental strain. On the contrary, Δ*omp31*, Δ*omp25c*-k, Δ*omp10*, Δ*omp19*, Δ*cgs*, and the remaining multiple mutants showed, in different degrees, lower CFU values than *B. ovis* PA (**Table 1**). The Δ*omp31*Δ*cgs* double mutant and its derived triple mutants, the Δ*omp25*Δ*cgs* mutant and the Δ*omp10*Δ*omp31*Δ*omp25c* and Δ*omp31*Δ*omp10*Δ*omp25c* triple mutants (bearing the same three genes inactivated but in a different order) showed the lowest CFU/ml values in TSA-YE-HS plates (**Table 1**). The numbers of CFU/ml obtained for each mutant were used for the calculation of the bacterial doses in further experiments.

**FIGURE 1:**
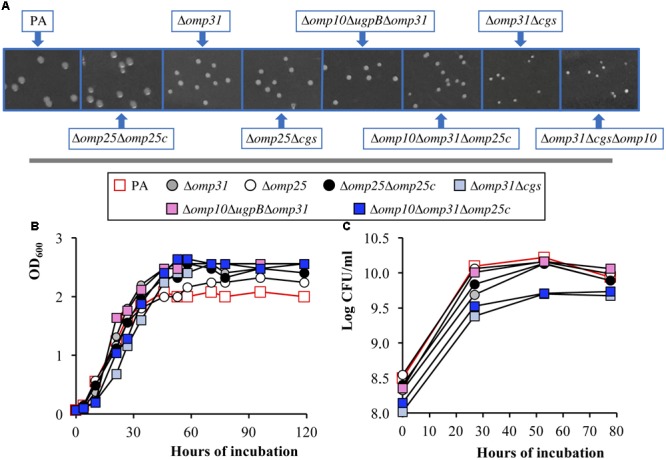
Growth pattern of *B. ovis* PA and selected mutants in TSA-YE-HS **(A)** and TSB-YE-HS **(B,C)**. Colony size in TSA-YE-HS **(A)** was photographed 5 days after inoculation. Plates were taken in the same picture and a section of each strain was extracted to compose the final panel. Growth in TSB-YE-HS liquid medium was determined by the evolution with time of the OD_600_ values **(B)** and of the bacterial CFU/ml numbers **(C)**. The SD of three assays, which was always lower than 5% of the mean, is not shown. To simplify the figure, relevant statistically significant differences (*P* < 0.05) are mentioned in the text.

In addition, the size of the colonies in TSA-YE-HS was monitored over time and recorded 5 days after inoculation (see some representative results in **Figure 1A**). Colony size of the Δ*omp25*, Δ*bacA*, Δ*bepC*, Δ*ugpB*, Δ*omp25c*-k, Δ*bepC*Δ*ugpB*, and Δ*omp25*Δ*omp25c* mutants was undistinguishable from that of the parental strain (see *B. ovis* PA and the Δ*omp25*Δ*omp25c* mutant in **Figure 1A**). Growth of the Δ*omp10* and Δ*omp19* mutants was slightly delayed when compared to the parental strain but the initial differences in colony size were not apparent after 5 days of incubation (data not shown). Colonies of the Δ*omp31* and Δ*cgs* single mutants appeared hardly visible 72 h after inoculation, 24 h later than those of the *B. ovis* PA parental strain, and were smaller than those of the parental strain after 5 days (see Δ*omp31* in **Figure 1A**). A similar or higher delay, depending on the strain, was detected with colonies of their multiple mutants. Despite the triple gene deletion, the Δ*omp10*Δ*ugpB*Δ*omp31* mutant showed a colony growth pattern undistinguishable from that of the single Δ*omp31* mutant (**Figure 1A**). The most important growth defects were observed with the Δ*omp31*Δ*cgs* mutant and its derived triple mutants (with the additional deletion of *omp10* or *omp19*), whose colonies started to be detected 96 h after inoculation and showed the smallest size after 5 days of incubation (**Figure 1A**). Colonies of the Δ*omp10*Δ*omp31*Δ*omp25c* and Δ*omp31*Δ*omp10*Δ*omp25c* triple mutants were also detected after 96 h of incubation but, at day 5 post-inoculation, they were bigger (see colonies of the Δ*omp10*Δ*omp31*Δ*omp25c* mutant that are representative for both mutants) than those of *B. ovis* Δ*omp31*Δ*cgs* (**Figure 1A**).

The Δ*omp31*Δ*cgs*, Δ*omp10*Δ*omp31*Δ*omp25c*, and Δ*omp10*Δ*ugpB*Δ*omp31* mutants (selected according to the results obtained in the evaluation of virulence described below), the Δ*omp25*Δ*omp25c* mutant (selected because it lacks two major OMPs in *B. ovis* PA) and their corresponding single mutants were also analyzed regarding growth in TSB-YE-HS liquid medium (**Figures 1B,C**). Cultures were adjusted to OD_600_ values of 0.05 and incubated at 37°C under agitation (120 rpm) and a 5% CO_2_ atmosphere. As expected, according to the correlation OD_600_-CFU/ml in solid medium previously determined, values of CFU/ml at t0 for mutants with inactivated *omp31* or *cgs* were lower than those of *B. ovis* PA (*P* < 0.05), the Δ*omp31*Δ*cgs* and Δ*omp10*Δ*omp31*Δ*omp25c* mutants presenting the lowest values (*P* < 0.05) (**Figure 1C**). However, the CFU/ml values of the Δ*omp31* and Δ*omp10*Δ*ugpB*Δ*omp31* mutants at the beginning of the stationary phase (approximately t52) were similar to those of *B. ovis* PA (*P* > 0.05), which correlates with the higher OD_600_ scores observed with these mutants by this time (*P* < 0.05). Although the Δ*omp31*Δ*cgs* and Δ*omp10*Δ*omp31*Δ*omp25c* mutants, those showing the most important growth defects in solid medium (**Figure 1A**), also had higher OD_600_ values at this moment (*P* < 0.05), their CFU/ml counts never reached the maximum level of *B. ovis* PA (detected at t52) (**Figures 1B,C**). The Δ*cgs*, Δ*omp25*, Δ*omp25c-k*, and Δ*omp25*Δ*omp25c* mutants had slightly higher OD_600_ scores in the stationary phase, when compared to the parental strain, but not important differences in the CFU/ml values (**Figures 1B,C** and data not shown). The Δ*omp10* and Δ*ugpB* mutants behaved similarly to the parental strain (data not shown).

### Virulence in the Mouse Model

For a preliminary assay of virulence, aiming to select the most relevant mutants, mice were inoculated with 10^6^ CFU of the parental strain or its derived mutants and bacteria were enumerated in spleen at W3 and W7 p.i. The multiple *cgs* mutants were excluded from this first analysis, since the single Δ*cgs* mutant showed poor spleen colonization in a previous study (Martín-Martín et al., 2012). Only those mutants that, when compared to the parental strain, showed bacterial counts lower than 1.5 logarithmic units were considered as probably attenuated and they were later inoculated in a dose of 10^8^ CFU/mouse. Uniquely the Δ*cgs*, Δ*omp10*Δ*ugpB*Δ*omp31*, Δ*omp10*Δ*omp31*Δ*omp25c*, and Δ*omp31*Δ*omp10*Δ*omp25c* mutants met this requirement and, together with the Δ*cgs* multiple mutants, were evaluated with the increased dose (except *B. ovis* Δ*omp31*Δ*omp10*Δ*omp25c* that bears the same mutations as the Δ*omp10*Δ*omp31*Δ*omp25c* but in a different order). All these mutants were present in spleen at W3 p.i. but the CFU were between 1 and 2.5 log units lower than those obtained with the parental strain (**Table 1**). All *cgs* mutants and *B. ovis* Δ*omp10*Δ*omp31*Δ*omp25c* had similar splenic counts at W7 p.i., which were not drastically different from those observed at W3 p.i., while the Δ*omp10*Δ*ugpB*Δ*omp31* mutant was not detected in spleen at W7 p.i. (**Table 1**).

**FIGURE 2:**
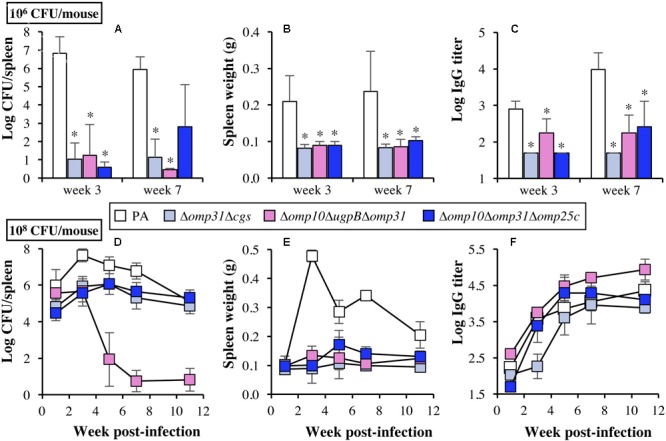
Bacterial spleen colonization **(A,D)**, spleen weight **(B,E)**, and IgG response in serum against *B. ovis* PA whole cells **(C,F)** in BALB/c mice. Mice (*n* = 5 per group and time point) were inoculated intraperitoneally with 10^6^ (upper panels) or 10^8^ (lower panels) CFU/mouse of *B. ovis* PA or Δ*omp31*Δ*cgs*, Δ*omp10*Δ*ugpB*Δ*omp31*, and Δ*omp10*Δ*omp31*Δ*omp25c* mutants. Statistically significant differences in upper panels, compared to parental *B. ovis* PA, are marked with an asterisk. To simplify the figure, relevant statistically significant differences in lower panels (*P* < 0.05) are mentioned in the text. Detection limit for antibody titers is log 1.7. As a reference for panels **B** and **E**, spleen weight of mice inoculated with PBS (*n* = 5) for the protection experiment described in **Figure 8** was 0.096 ± 0.005 (W7 post-inoculation).

According to these results, the Δ*omp31*Δ*cgs*, Δ*omp10* Δ*ugpB*Δ*omp31*, and Δ*omp10*Δ*omp31*Δ*omp25c* mutants were retained for a detailed evaluation of virulence and the selection of vaccine candidates. First, to statistically verify the previous results, the spleen colonization was evaluated in five mice per group at W3 and W7 after intraperitoneal infection with 10^6^ CFU (**Figure 2A**). The spleen weight (**Figure 2B**) and the levels in serum of IgG reacting against *B. ovis* PA whole cells (**Figure 2C**) were also determined. As expected, spleen infection of *B. ovis* PA was noteworthy at both sampling points – with mean values ranging between log 6 and 7 of CFU/spleen – while the three mutants presented a poor colonization under these conditions (**Figure 2A**). Spleen infection of the mutants was undetectable in several mice per group, although two out five mice inoculated with the Δ*omp10*Δ*omp31*Δ*omp25c* mutant gave values of about log 5 CFU/spleen at W7 p.i. (**Figure 2A**). Spleen weight and IgG titers in serum were also significantly higher (*P* < 0.05) in mice inoculated with the parental strain (**Figures 2B,C**).

The same parameters described above were evaluated at several time points over a 11-week period in mice inoculated with 10^8^ CFU of each strain (**Figures 2D–F**), the dose usually employed for evaluation of attenuated strains as vaccines (Sancho et al., 2014; Soler-Lloréns et al., 2014; Silva et al., 2015b). The parental strain showed high levels of spleen infection through the entire experiment with mean log CFU/spleen values of 5.99 at W1 p.i. and a peak at W3 p.i. of 7.64. *B. ovis* PA counts decreased thereafter but were still high at the end of the experiment (mean log CFU/spleen values of 5.01 at W11) (**Figure 2D**). The Δ*omp31*Δ*cgs* and Δ*omp10*Δ*omp31*Δ*omp25c* mutants displayed lower levels of splenic infection at W1, with mean CFU/spleen values 1–1.5 log lower than those of the parental strain (*P* < 0.05). However, although their maximum CFU/spleen scores (about log 6 at W3–W5 p.i.) did never reach those of *B. ovis* PA (log 7.64 at W3 p.i.), they persisted in spleen with similar values to those of the parental strain at W11 p.i. (*P* > 0.05) (**Figure 2D**). The spleen colonization of the Δ*omp10*Δ*ugpB*Δ*omp31* mutant did not show statistically significant differences with the parental strain at W1 p.i., but the bacterial counts did not increase at W3 p.i. and decreased thereafter until the detection limit of infection at W7 p.i. (*P* < 0.05) (**Figure 2D**).

At W1 p.i., the spleen weight in mice infected with 10^8^ CFU of the parental strain (0.102 ± 0.030 g) was analogous to that currently obtained with mice inoculated with PBS and that was also observed in the PBS group used to determine the production of cytokines by splenocytes in the experiment described below (0.096 ± 0.005 g) (*P* > 0.05). However, a prominent splenomegaly was detected at W3 p.i. (0.478 ± 0.020 g/spleen) which, although decreased after W3 p.i., persisted until the end of the experiment (0.205 ± 0.046 g/spleen) (*P* < 0.05) (**Figure 2E**). On the contrary, the *B. ovis* mutants did never reach mean values of 0.200 g/spleen (**Figure 2E**). At W1 p.i., all groups of mice had low levels of IgG reactive with whole cells of *B. ovis* PA. An important increase of the antibody response was detected at W3 p.i. in all groups (*P* < 0.05), except in mice vaccinated with the Δ*omp31*Δ*cgs* mutant (*P* > 0.05). At W5 p.i. and thereafter, mice inoculated with the parental strain or the Δ*omp31*Δ*cgs* and Δ*omp10*Δ*omp31*Δ*omp25c* mutants displayed IgG titers of about log 4, while mice inoculated with *B. ovis* Δ*omp10*Δ*ugpB*Δ*omp31* gave titers in the order of 0.5 logarithmic units higher (*P* < 0.05) than those detected in the *B. ovis* PA group (**Figure 2F**).

### Autoagglutination and Susceptibility Assays

**FIGURE 3:**
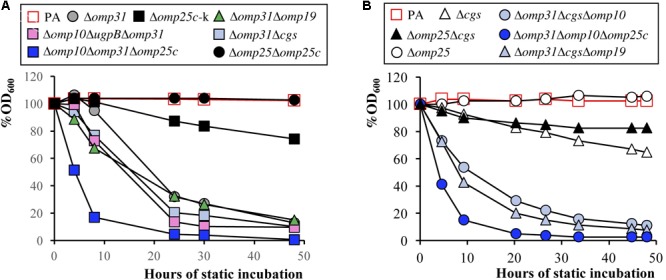
Autoagglutination assay with bacterial suspensions of initial OD_600_ readings of 0.8 (100% OD_600_). The percentage of the OD_600_ values was determined over 48 h of static incubation. Panels **A** and **B** represent results of two independent experiments. The SD of three assays, which was always lower than 5% of the mean, is not shown. To simplify the figure, relevant statistically significant differences (*P* < 0.05) are mentioned in the text.

In addition to the three selected mutants, other relevant mutants were included in the remaining studies by their interest to increase the knowledge about the *B. ovis* cell envelope. Several tests related to the OM properties of *Brucella* spp. (i.e., autoagglutination and resistance to polymyxin B and sodium deoxycholate) (Martín-Martín et al., 2011; Vizcaíno and Cloeckaert, 2012) were performed. In the autoagglutination assay, most of the single mutants behaved as the *B. ovis* PA parental strain (*P* > 0.05), remaining in suspension until the end of the experiment (**Figure 3** and data not shown). The exceptions were *B. ovis* Δ*omp31* – with %OD_600_ values of about 30 and 10% after 24 and 48 h of static incubation, respectively – and the Δ*cgs* and Δ*omp25c*-k mutants, whose %OD_600_ values gradually decreased until 70% at the end of the experiment (*P* < 0.05 when compared to the parental strain) (**Figure 3**). The *omp31* multiple mutants behaved similarly to the single mutant, although the Δ*omp10*Δ*omp31*Δ*omp25c* and Δ*omp31*Δ*omp10*Δ*omp25c* mutants agglutinated more quickly (they were almost completely settled after 10 h) (*P* < 0.05) (**Figure 3**). *B. ovis* Δ*omp25*Δ*cgs* behaved like the single Δ*cgs* mutant while *B. ovis* Δ*omp25*Δ*omp25c* did not show differences with the parental strain (*P* > 0.05) (**Figure 3**).

*Brucella ovis* Δ*bacA* and the *B. ovis* Δ*bacA*Δ*omp31* double mutant were more resistant than *B. ovis* PA to polymyxin B exposure for 80 min (**Table 4**). On the contrary, *B. ovis* Δ*omp25*Δ*omp25c*, and the Δ*omp31* single and multiple mutants – except the previously mentioned Δ*bacA*Δ*omp31* mutant – showed higher susceptibility to polymyxin B than the parental strain (**Table 4**). The lowest survival percentages were obtained with the Δ*omp10*Δ*omp31*Δ*omp25c* and Δ*omp31*Δ*omp10*Δ*omp25c* mutants (less than 10% survival). Only the Δ*omp31*Δ*cgs* double mutant and its derived Δ*omp31*Δ*cgs*Δ*omp10* and Δ*omp31*Δ*cgs*Δ*omp19* triple mutants were more susceptible to sodium deoxycholate than the parental strain (about 30% survival versus 86% survival, respectively) (**Table 4**).

**Table 4:** Susceptibility of *B. ovis* PA and selected mutants to polymyxin B and Na deoxycholate.

	% Survival after exposure to^a^:	
*Brucella ovis* strains^b^	Polymyxin B (1 mg/ml)	Na deoxycholate (0.1 mg/ml)
*B. ovis* PA	69.03 6.12	86.25 5.74
**Single mutants**		
Δ*omp31*	27.47 3.04*	83.74 3.09
Δ*omp25*	65.84 7.17	92.94 4.06
Δ*omp25c*-k	65.99 4.72	95.89 7.15
Δ*cgs*	77.96 11.80	79.82 9.07
Δ*bacA*	84.10 5.49*	87.38 8.28
Δ*omp10*	62.04 3.46	91.20 4.22
Δ*omp19*	67.27 7.05	83.19 7.75
Δ*ugpB*	77.59 11.67	90.66 9.42
**Double mutants**		
Δ*omp31*Δ*omp19*	25.68 2.94*	90.49 7.79
Δ*omp31*Δ*ugpB*	20.66 0.57*	92.65 3.64
Δ*omp31*Δ*cgs*	31.20 14.30*	32.14 1.27*
Δ*omp25*Δ*cgs*	61.71 7.30	92.35 5.18
Δ*omp25*Δ*omp25c*	45.23 5.10*	91.36 9.59
Δ*omp10*Δ*omp31*	33.31 4.58*	84.66 6.36
Δ*omp10*Δ*ugpB*	64.22 5.36	77.93 9.26
Δ*bacA*Δ*omp31*	88.06 18.61*	86.90 5.51
**Triple mutants**		
Δ*omp10*Δ*ugpB*Δ*omp31*	30.50 9.94*	88.52 6.22
Δ*omp10*Δ*omp31*Δ*omp25c*	1.65 0.36*	76.74 9.37
Δ*omp31*Δ*omp10*Δ*omp25c*	7.19 4.38*	80.40 7.49
Δ*omp31*Δ*cgs*Δ*omp10*	37.84 9.43*	33.63 1.78*
Δ*omp31*Δ*cgs*Δ*omp19*	33.30 3.68*	34.39 2.94*

*^*a*^Results are expressed as the mean ± SD of three assays. Statistically significant differences (*P* < 0.05), compared to the parental strain, are marked with an asterisk. ^*b*^See legend to **Figure 1** for strain characteristics.*

### Mapping of Cell Envelope Antigens

**FIGURE 4:**
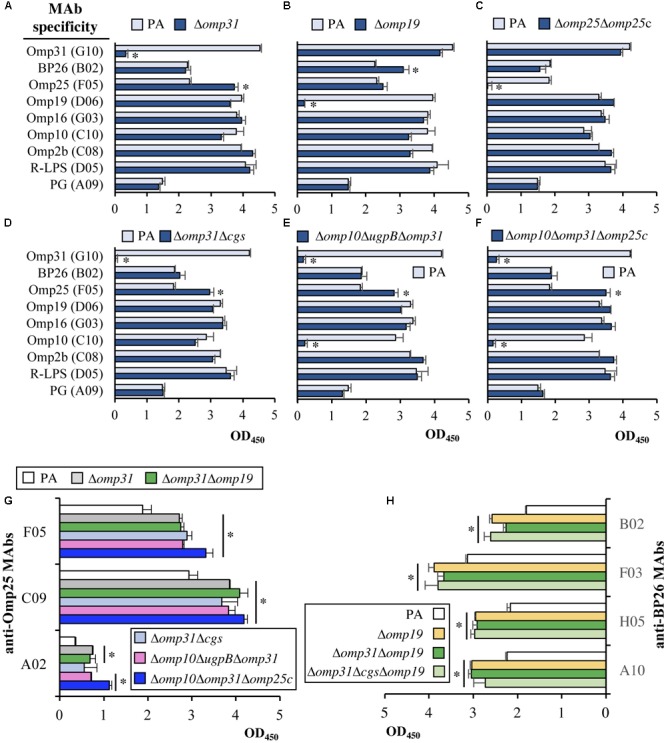
Reactivity by iELISA of MAbs against cell envelope antigens with *B. ovis* PA and selected mutants. The reactivity of MAbs specific against PG, R-LPS, the BP26 periplasmic protein, the Omp2b, Omp31, and Omp25 major OMPS, and the Omp10, Omp16, and Omp19 OM lipoproteins **(A–F)** was tested with most of the mutant strains obtained in this work. Representative results are shown in panels **A–F** and others are commented in the text. Differences between the mutant strains and the parental strain were considered relevant for further analysis when, in addition to being statistically significant (*P* < 0.05), the mean values differed more than 25% (marked with an asterisk). Reactivity of Δ*omp31* single and multiple mutants with several anti-Omp25 MAbs **(G)**, and of Δ*omp19* mutants with several anti-BP26 MAbs **(H)**, is also shown. Statistically significant differences between the mutant strains and the parental strain (*P* < 0.05) in panels **G** and **H**, are marked with an asterisk.

The reactivity of the *B. ovis* mutants with MAbs raised against *Brucella* S-LPS, R-LPS, PG, major and minor OMPs, and periplasmic BP26 was analyzed by iELISA (**Figure 4** and data not shown). As expected, the MAb-specific against S-LPS did not react with any mutant or with the parental strain (data not shown) and *omp31, omp25, omp10*, or *omp19* single and multiple mutants did not react with the respective MAbs (see **Figure 4** for representative results). No relevant differences were observed between the parental strain and the mutants regarding reactivity with MAbs specific against Omp31 (except with mutants lacking *omp31*), Omp10 (except with mutants lacking *omp10*), Omp16, Omp2b, PG, and R-LPS. On the contrary, all *omp31* mutants showed a stronger reaction with MAb C09 (specific against Omp25) and *omp19* mutants showed a stronger reaction with MAb B02, specific against BP26 (**Figure 4**, upper panels and data not shown). Accordingly, several *omp31* mutants were tested in iELISA with three MAbs specific against Omp25, while *omp19* mutants were tested with four anti-BP26 MAbs. All the Δ*omp31* and Δ*omp19* mutants showed a stronger reactivity with the anti-Omp25 and anti-BP26 MAbs, respectively (**Figures 4G,H**), which confirmed the results obtained in the previous analysis.

**FIGURE 5:**
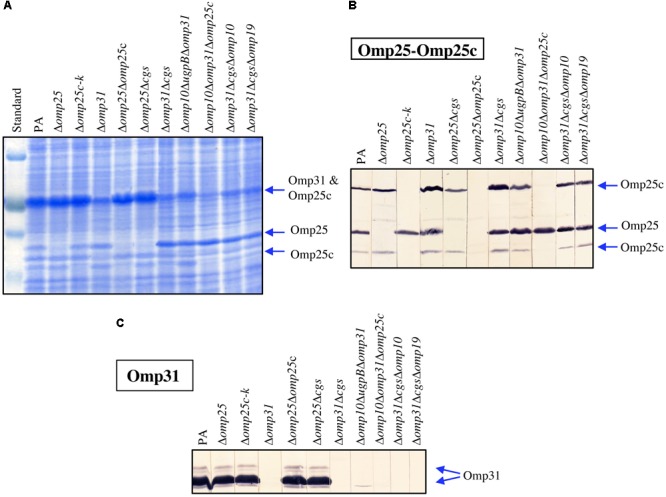
Profiles of *B. ovis* PA and selected mutants in SDS–PAGE **(A)** and immunoblot with sera of rabbits immunized with purified recombinant Omp25c **(B)** or Omp31b **(C)**. The prestained protein marker VI (Applichem-Panreac) is shown in **(A)** and positions of known OMP bands are marked with arrows **(A–C)**.

The protein profile of the most relevant strains was evaluated by SDS–PAGE followed by Coomassie blue straining (**Figure 5A**). Proteins were also transferred to nitrocellulose to assess the reactivity with sera raised against the proteins of the Omp25/Omp31 family. A serum against Omp25c was used to detect both Omp25c and Omp25 since these proteins display cross-reacting epitopes (Martín-Martín et al., 2009), while the detection of Omp31 was performed with a serum raised against Omp31b (an OMP absent in *B. ovis*) that strongly cross-react with Omp31 (Martín-Martín et al., 2009). Omp25d and Omp22 were detected by reactivity with their respective anti-sera (Martín-Martín et al., 2009).

A multiple band pattern, dependent on the electrophoretic conditions, is frequently observed in SDS–PAGE for Omp25, Omp25c, and Omp31 (Cloeckaert et al., 1990; Martín-Martín et al., 2009). Two protein bands corresponding to Omp25c were detected by immunoblot in parental *B. ovis* PA and were absent in all *omp25c* mutants (**Figure 5B**). Additionally, the intensity of the upper band in *omp31* mutants was higher than that observed with the parental strain (**Figure 5B**). Absence of the lower band of Omp25c in the Δ*omp25c* mutants was also evident in the SDS–PAGE gel (**Figure 5A**, lanes Δ*omp25c-k*, Δ*omp25Domp25c*, and Δ*omp10*Δ*omp31*Δ*omp25c*) while the absence of the upper band was only apparent in the Δ*omp10*Δ*omp31*Δ*omp25c* triple mutant (**Figure 5A**). This result can be explained by the fact that this mutant also lacks Omp31, which is a major OMP that exhibits an electrophoretic mobility similar to that of Omp25c (**Figure 5A**) and, consequently, masks the absence of the upper Omp25c band in the other Δ*omp25c* mutants.

Omp25 was detected between the two Omp25c bands except in the *omp25* mutants. Absence of Omp25 in these mutants was evidenced in both the SDS–PAGE gel (**Figure 5A**) and the immunostained nitrocellulose (**Figure 5B**) (see Δ*omp25*, Δ*omp25*Δ*cgs*, and Δ*omp25*Δ*omp25c* lanes in comparison with PA lanes). With both techniques, the Omp25 band of the Δ*omp31* mutants was more intense than in *B. ovis* PA. Omp31 was detected in immunoblot and with the same intensity in all the strains, except those lacking the encoding gene (**Figure 5C**). Absence of Omp31 in these latter mutants was also evident in the SDS–PAGE gel (**Figure 5A**). The Omp22 band was revealed in *B. ovis* PA and the analyzed mutants, while Omp25d – that has only been observed in a complemented mutant overexpressing the protein (Martín-Martín et al., 2009) – was not detected (data not shown).

### Cellular Models of Infection

**FIGURE 6:**
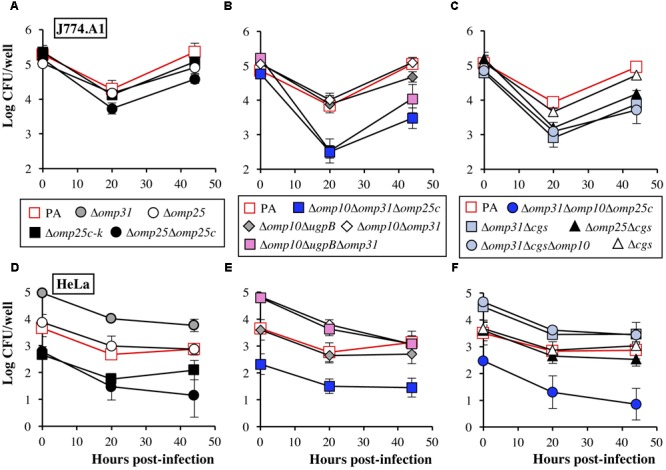
Internalization and intracellular behavior of *B. ovis* PA and selected mutants in J774.A1 murine macrophages **(A–C)** and HeLa cells **(D–F)**. The results are expressed as the means ± SD (*n* = 3) of the log CFU/well at each time point. To simplify the figure, relevant statistically significant differences (*P* < 0.05) are mentioned in the text.

The intracellular behavior of *B. ovis* PA and the most relevant mutants was evaluated in J774.A1 murine macrophages and HeLa cells. No significant differences between strains (*P* > 0.05) were observed regarding internalization in J774.A1 macrophages (**Figure 6**, upper panels). However, although the intracellular bacterial numbers decreased at t20 with all the strains, the reduction was more pronounced with strains Δ*omp25*Δ*omp25c* (**Figure 6A**), Δ*omp25*Δ*cgs*, Δ*omp31*Δ*cgs*, and its derived triple mutants (**Figure 6C**), and with *B. ovis* Δ*omp10*Δ*ugpB*Δ*omp31* and Δ*omp10*Δ*omp31*Δ*omp25c* (**Figure 6B**) (*P* < 0.05). After this moment, all strains were able to replicate, reaching intracellular CFU numbers at t44 about 1 log unit higher than those detected at t20 for each strain (*P* < 0.05) (**Figure 6**, upper panels).

In HeLa cells, the Δ*omp25c* mutants (Δ*omp25c*-k, Δ*omp25*Δ*omp25c*, Δ*omp10*Δ*omp31*Δ*omp25c*, and Δ*omp31* Δ*omp10*Δ*omp25c*) showed a deficient internalization, with intracellular CFU at t0 in the order of 1–1.5 log units lower than those determined for *B. ovis* PA (*P* < 0.05). On the contrary, all mutants bearing the *omp31* deletion, except those with the Δ*omp25c* genotype (*B. ovis* Δ*omp10*Δ*omp31*Δ*omp25c* and Δ*omp31*Δ*omp10*Δ*omp25c*), showed an increased internalization in HeLa cells, with CFU at t0 about 1 log higher than those of the parental strain (*P* < 0.05) (**Figure 6**, lower panels). All strains suffered a reduction (*P* < 0.05) of intracellular CFU a t20 while at t44 the bacterial numbers were, in general, similar to those obtained at t20 (**Figure 6**, lower panels).

**FIGURE 7:**
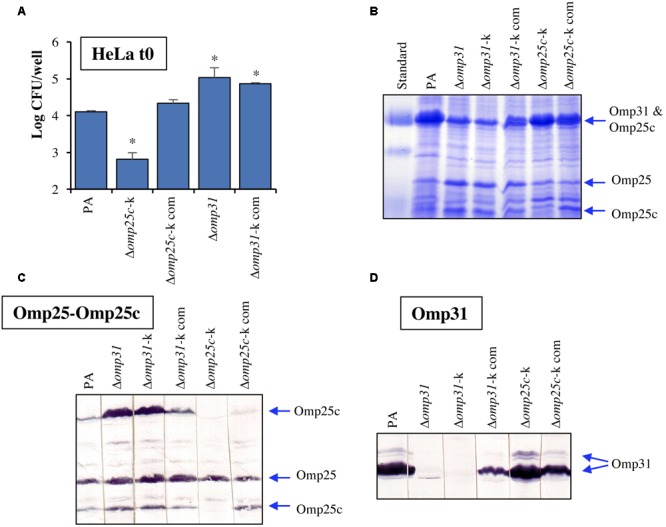
**(A)** Internalization in HeLa cells of parental *B. ovis* PA, the Δ*omp25c* and Δ*omp31* mutants and mutant strains complemented with wild-type *omp25c* or *omp31*. SDS–PAGE profiles **(B)** and immunoblot detection of Omp25, Omp25c **(C)**, and Omp31 **(D)** in the same strains. Strains Δ*omp31*-k and Δ*omp25c*-k were previously obtained by replacement of *omp31* or *omp25c* by a kanamycin-resistance cassette (Caro-Hernández et al., 2007). They were complemented with the corresponding wild-type genes to give strains identified as Δ*omp31*-k com and Δ*omp25c*-k com, respectively (Caro-Hernández et al., 2007). The prestained protein marker VI (Applichem-Panreac) is shown in **(B)** and positions of known OMP bands are marked with arrows. Statistically significant differences when compared to the parental strain are marked with an asterisk.

Searching for an explanation to the internalization differences observed in HeLa cells, the Δ*omp25c*-k mutant complemented with the wild-type gene and the Δ*omp31*-k mutant together with its complemented strain (Caro-Hernández et al., 2007) were also analyzed regarding internalization in HeLa cells (CFU determined at t0) and their behavior in immunoblot with sera reacting with Omp31 and Omp25c (**Figure 7**). Complementation of the Δ*omp25c*-k mutant restored its ability to enter HeLa cells like the parental strain (**Figure 7A**) and the production of Omp25c (**Figures 6B,C**). The complemented Δ*omp31*-k mutant recovered the ability to produce Omp31, but its level was lower than that of the parental strain (**Figures 7B,D**). This fact was concomitant with a higher intensity of Omp25c bands, when compared to *B. ovis* PA that, however, did not reach the intensity observed with the Δ*omp31* and Δ*omp31*-k mutants (**Figure 7C**). Internalization of the complemented Δ*omp31*-k mutant in HeLa cells was similar to that of the Δ*omp31* mutant and significantly higher than that observed with the parental strain (**Figure 7A**).

### Efficacy as Vaccine of *B. ovis* Δ*omp10*Δ*ugpB*Δ*omp31*

**FIGURE 8:**
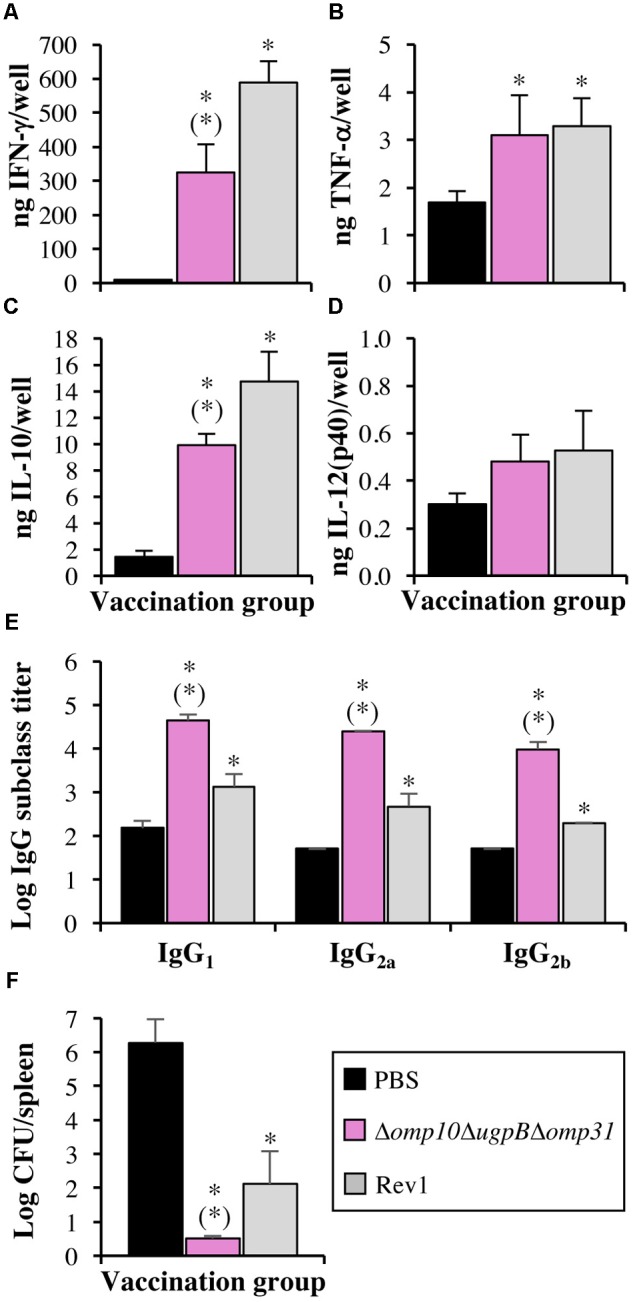
Cytokine secretion by stimulated splenocytes **(A–D)**, levels in serum of IgG subclasses reactive with *B. ovis* PA whole cells **(E)**, and protective efficacy against *B. ovis* PA infection **(F)** in mice vaccinated with *B. ovis* Δ*omp10*Δ*ugpB*Δ*omp31* or *B. melitensis* Rev1 and in mice inoculated with PBS. Splenocytes **(A–D)** and sera **(E)** were obtained 7 weeks after inoculation (five mice per group). At that time, five mice per group were inoculated with virulent *B. ovis* PA and bacterial colonization of spleen was determined 3 weeks later to evaluate the protective efficacy of the vaccines **(F)**. Splenocytes were stimulated with heat-killed *B. ovis* PA whole cells for 72 h **(A–D)** before the evaluation of secreted cytokines. Statistically significant differences, compared to mice inoculated with PBS, are marked with an asterisk. Statistically significant differences of the Δ*omp10*Δ*ugpB*Δ*omp31* group compared to the *B. melitensis* Rev1 group are marked with an asterisk in brackets.

The usefulness as vaccine of *B. ovis* Δ*omp10*Δ*ugpB*Δ*omp31* against *B. ovis* PA was evaluated in mice and compared to that of the *B. melitensis* Rev1 heterologous vaccine. Spleen colonization of the challenge strain was evaluated 3 weeks after infection (**Figure 8F**) and the humoral (**Figure 8E**) and cellular (**Figures 8A–D**) immune response was evaluated at the time point selected for the challenge with the virulent strain (W7 post-vaccination).

Weight of spleens obtained at W7 post-vaccination from mice inoculated with the Δ*omp10*Δ*ugpB*Δ*omp31* mutant (0.104 ± 0.012 g) did not show statistically significant differences with that of mice inoculated with PBS (0.096 ± 0.005 g), while splenomegaly (*P* < 0.05) was detected in mice vaccinated with *B. melitensis* Rev1 (0.136 ± 0.011 g) (data not shown). Additionally, *B. melitensis* Rev1 was detected in spleen (log CFU/spleen values of 3.66 ± 0.07), while the Δ*omp10*Δ*ugpB*Δ*omp31* mutant was cleared by this time (data not shown).

Splenocytes were stimulated with *B. ovis* PA whole cells to evaluate, by specific ELISA tests, the production of cytokines. Splenocytes of mice inoculated with PBS secreted limited amounts of IFN-γ, while about 30 and 60 times higher levels (*P* < 0.05) were detected in the groups vaccinated with the Δ*omp10*Δ*ugpB*Δ*omp31* mutant and *B. melitensis* Rev1, respectively (**Figure 8A**). In a lesser extent, splenocytes from vaccinated mice also produced more IL-10 (*P* < 0.05) than those obtained from mice of the PBS group (mean values of 14.7, 9.9, and 1.4 ng/well for the *B. melitensis* Rev1, *B. ovis* Δ*omp10*Δ*ugpB*Δ*omp31*, and PBS groups, respectively) (**Figure 8C**). Regarding the production of TNF-α, splenocytes from mice vaccinated with the two *Brucella* attenuated strains secreted about double amounts (mean values of 3 ng/well) than those obtained from the unvaccinated control group (*P* < 0.05) (**Figure 8B**). No statistically significant differences were observed between groups concerning the production of IL-12(p40) (**Figure 8D**).

At the time of challenge, mice inoculated with PBS 7 weeks earlier showed low titers (close to the detection limit of log 1.7) of serum antibodies of the IgG_1_, IgG_2a_, and IgG_2b_ subclasses able to react with *B. ovis* PA whole cells in iELISA (**Figure 8E**). On the contrary, mice vaccinated with *B. ovis* Δ*omp10*Δ*ugpB*Δ*omp31* had titers of the three IgG subclasses (ranging from log 3.99 ± 0.16 for IgG_2b_ to log 4.65 ± 0.13 for IgG_1_) that were about 1.5 log units higher (*P* < 0.05) than those detected in mice vaccinated with the *B. melitensis* Rev1 heterologous vaccine. Within each vaccination group, no relevant differences were observed between the three IgG subclasses (**Figure 8E**).

The homologous *B. ovis* Δ*omp10*Δ*ugpB*Δ*omp31* vaccine was not detected in spleen 3 weeks after the experimental challenge while it prevented spleen colonization of virulent *B. ovis* PA (CFU/spleen counts were under the detection limit) (**Figure 8F**). When compared to control mice inoculated with PBS, vaccination with heterologous *B. melitensis* Rev1 also drastically reduced the spleen colonization of *B. ovis* PA (**Figure 8F**), but both the vaccine strain and the challenge strain were detected in some mice.

## Discussion

With the aim to increase our knowledge about the OM and virulence of *B. ovis* and to develop a specific homologous attenuated vaccine, multiple deletion mutants targeting nine genes related to the OM were obtained and characterized. Two of these genes encode Omp10 and Omp19, two lipoproteins that are conserved in *Brucella* (Cloeckaert et al., 1990; Tibor et al., 1994, 1996) and have orthologs in other phylogenetically related bacteria able to establish interactions with eukaryotic cells either as symbionts or as pathogens (e.g., *Ochrobactrum, Bartonella, Rhizobium, Ensifer, Sinorhizobium*) (Cloeckaert et al., 1999; Barquero-Calvo et al., 2009). This observation suggests their relevant role for the bacterium and, in fact, both lipoproteins have been linked to the virulence of *B. abortus* 544 (Tibor et al., 2002). Surprisingly, the individual absence of Omp10 and Omp19 in the OM of *B. ovis* PA did not affect its virulence or modified notably its OM-related properties (Sidhu-Muñoz et al., 2016). On the contrary, the multiple assays we have performed to achieve the simultaneous deletion of *omp10* and *omp19* in *B. ovis* PA were unsuccessful. Since OM lipoproteins are thought to anchor the OM through the lipid moiety and contribute to the stability of the cell envelope (Goolab et al., 2015), Omp10 and Omp19 could have an interchangeable role necessary to maintain the OM integrity. Nevertheless, the amount of Omp10 in *B. ovis* Δ*omp19* and the amount of Omp19 in the Δ*omp10* mutant does not seem to be increased (**Figure 4B** and data not shown), although the absence of one of these lipoproteins, in the presence of the other, could also be compensated by an increase in other cell envelope proteins. In this respect, a higher reactivity with MAbs against BP26 suggestive of increased levels of this periplasmic protein was detected in the mutants bearing the *omp19* deletion (**Figure 4H**). Contrasting with the impossibility to obtain the double Δ*omp10*Δ*omp19* mutant, the simultaneous absence of major Omp31 together with Omp10 or Omp19 lipoprotein was well tolerated in *B. ovis* PA, since the respective double mutants do not have prominent defects in virulence (**Table 1**) and the other phenotypic changes are associated to the deletion of *omp31* (**Table 4, Figures 3, 5, 6**, and data not shown). Similarly, deletion of *ugpB* on the Δ*omp10* or Δ*omp19* genetic background did not affect the virulence (**Table 1**), and the alterations detected after deletion of *cgs* on the same mutants resembled those detected with the *B. ovis* Δ*cgs* single mutant (**Table 1**).

Other target surface antigens were Omp31, Omp25, and Omp25c that are abundant OMPs in *B. ovis* (Cloeckaert et al., 2002; Martín-Martín et al., 2009; Vizcaíno and Cloeckaert, 2012) and the main members of the Omp25/Omp31 family that is constituted by seven homologous OMPs (Salhi et al., 2003; Vizcaíno et al., 2004; Vizcaíno and Cloeckaert, 2012). Orthologs of this family of OMPs have been found in *Bartonella quintana* (Minnick et al., 2003) and other Rhizobiales (e.g., *Rhizobium leguminosarum, Agrobacterium tumefaciens*, or *O. anthropi*), which suggests that the redundancy of these OMPs provides an advantage that could be related with the compensatory effect between these proteins previously reported (Salhi et al., 2003; Caro-Hernández et al., 2007; Martín-Martín et al., 2009; Vizcaíno and Cloeckaert, 2012). This compensatory effect is in accordance with the apparent increase of Omp25 and Omp25c that was observed with all *omp31* mutants, except those also lacking *omp25c* where Omp25c was not detected (**Figures 4, 5**). On the contrary, *omp25* and *omp25c* mutants and even the double Δ*omp25*Δ*omp25c* mutant did not show higher levels of Omp31 (**Figures 4, 5**). In addition, this latter double mutant did not suffer important defects regarding *in vitro* growth (**Figure 1**) or virulence in mice (**Table 1**). These observations could be indicative of a more relevant role for Omp31, whose absence in the OM would require a compensation with another paralog that would not be necessary when Omp25 or Omp25c are missing.

However, considering that our attempts to obtain a *B. ovis* PA double mutant defective in Omp31 and Omp25 were fruitless, it seems probable that the presence in the bacterial surface of one of these two major OMPs is a requirement at least for *in vitro* survival. This assertion is in contradiction with the availability of Δ*omp25* mutants in *B. abortus* (Edmonds et al., 2002; Manterola et al., 2007), a *Brucella* species naturally lacking Omp31 due to a 25-kb deletion involving its encoding gene (Vizcaíno et al., 2001a). Nevertheless, unlike *B. ovis* where *omp31b* is a pseudogene (Vizcaíno et al., 2004), *B. abortus* strains synthetize Omp31b (Martín-Martín et al., 2009), an OMP sharing 67% of amino acid identity with Omp31 (Salhi et al., 2003) and that could compensate for the absence of Omp31 in this species. Research aiming to construct the Δ*omp25*Δ*omp31b* double mutant in the *B. abortus* genetic background would help to a better understanding of the relationships between the members of the Omp25/Omp31 family.

According to the availability of the Δ*omp10*Δ*omp31*Δ*omp25c* and Δ*omp31*Δ*omp10*Δ*omp25c* mutants (with the same deleted genes in a different order), *B. ovis* PA survives to the simultaneous absence of Omp10 lipoprotein and major Omp31 and Omp25c, together with the natural lack of Omp31b and Omp25b (Martín-Martín et al., 2009). However, both mutants suffered important phenotypic changes, such as strong *in vitro* growth defects, quick autoagglutination, remarkable susceptibility to polymyxin B, increased killing by murine macrophages in the first 20 h of infection, defective internalization in HeLa cells, and limited spleen colonization in mice inoculated with 10^6^ CFU. Nevertheless, not only both strains replicate in murine macrophages after 20 h of infection (**Figure 6B** and data not shown) but at least *B. ovis* Δ*omp10*Δ*omp31*Δ*omp25c* can establish a chronic infection in mice, with important levels of spleen colonization, when inoculated at 10^8^ CFU/mouse (**Figure 2D**).

Another finding regarding the Omp25/Omp31 family, and that was further confirmed by confocal microscopy (data not shown), is that all *omp25c* mutants are impaired for internalization in HeLa cells (**Figures 6D–F**), while all strains bearing the Δ*omp31* mutation (except the triple mutants also bearing the *omp25c* deletion) internalize more efficiently than the parental strain (**Figures 6D–F** and data not shown). Since immunoblot assays showed that Δ*omp31* mutants had higher levels of Omp25c, we postulated that Omp25c is involved in the internalization of *B. ovis* PA in HeLa cells. This hypothesis is supported by the immunoblots and HeLa cells assays performed with *omp31* and *omp25c* mutant and complemented strains (**Figure 7**). Whether this characteristic is specific for HeLa cells, for human cells or for non-professional phagocytes independently of the animal species remains to be studied.

As expected (Martín-Martín et al., 2012) the Δ*cgs* mutant did not suffer drastic changes in the *in vitro* characteristics, but was the only single mutant that was attenuated in the murine model when it was inoculated at 10^6^ CFU/mouse (**Table 1**). However, a 100-fold increase of the inoculation dose led to high splenic infection levels at both the acute and chronic phase of infection (**Table 1**) and to a kinetics of spleen colonization resembling that observed in mice experimentally infected with 10^6^ CFU of the parental strain (data not shown; see the equivalent spleen colonization profile of the Δ*omp31*Δ*cgs* mutant in **Figure 2**). According to the similarities between the parental strain and the Δ*cgs* mutant regarding their behavior in the cellular models of professional and non-professional phagocytes used in this work (**Figures 6C,F**) the attenuation of the Δ*cgs* mutant of *B. ovis* PA does not seem to be due to killing inside phagocytic cells. However, since we have used individual cell lines, a defective interaction with one or more types of professional and/or non-professional phagocytes of BALB/c mice cannot be discarded. In fact, Δ*cgs* mutants of *B. abortus* 2308 and S19 are defective in both HeLa cells (Briones et al., 2001; Arellano-Reynoso et al., 2005; Roset et al., 2006, 2014) and peritoneal macrophages of C57BL/6 mice (Arellano-Reynoso et al., 2005) but, on the contrary, they behave as the parental strains in bone marrow cells also obtained from C57BL/6 mice (Salcedo et al., 2008; Roset et al., 2014). Another possible explanation for the attenuation of *B. ovis* Δ*cgs* could be related to the association that has been established between CβGs and a dual pro- and anti-inflammatory response that transiently recruits neutrophils and suggests a controlled local inflammatory response (Degos et al., 2015). This controlled inflammatory response might be important for the establishment of the *Brucella* infection, since survival and replication inside phagocytes is a characteristic trait of *Brucella* that provides the bacterium with a safe environment. A strong inflammatory process would trigger a detrimental immune response against the pathogen but, on the contrary, a diminished inflammatory response in the absence of CβGs would reduce the presence of suitable target cells for *Brucella* replication and, therefore, could influence the outcome of infection.

The Δ*omp31*Δ*cgs* double mutant, defective in a major OMP and in periplasmic CβGs, maintained or exacerbated the most severe phenotype of the corresponding single mutants. Thus, the susceptibility to DOC (**Table 4**) and *in vitro* growth defects were more prominent in the Δ*omp31*Δ*cgs* mutant than in the single mutants, which would be in accordance with cell envelope modifications compromising the permeability of the bacterial cell to nutrients and toxic compounds. Strikingly, despite its important *in vitro* growth impairment and its increased killing during the first stage of infection in murine macrophages (**Figure 6C**), the Δ*omp31*Δ*cgs* mutant behaved similarly to the Δ*cgs* mutant in the mouse model, except for a lower spleen colonization at W3 p.i. (**Table 1, Figure 2**, and data not shown). Additionally, a third deletion of *omp10* or *omp19* did not produce apparent differences with respect to the Δ*omp31*Δ*cgs* mutant in any of the characteristics evaluated and attenuation of the Δ*cgs* mutant was not drastically intensified by the deletion of *omp10*, o*mp19, bepC, ugpB*, or *omp25* (**Table 1**).

Similarly to *B. ovis* Δ*omp31*Δ*cgs*, the Δ*omp10*Δ*omp31* Δ*omp25c* mutant suffered a higher decrease of the intracellular CFU during the first 20 h of macrophage infection but both mutants multiplied properly after that moment (**Figures 6B,C**). This ability to replicate intracellularly might explain why, despite their impairment to infect mice at doses of 10^6^ CFU/mouse, they persisted at least until week 11 p.i. – with splenic counts equivalent to those of the parental strain at that moment – when 10^8^ CFU were inoculated (**Figure 2**). Increased killing inside professional phagocytes at the onset of infection could have dramatic effects on the outcome of infection when the lower dose of infection is used. However, higher doses of infection would allow some bacteria to escape to the bactericidal mechanisms of phagocytes and reach the replicative niche to establish a persistent infection thereafter. At least in part, the higher reduction of intracellular CFU at t20 could be related to a defective growth inside the phagocytes – mimicking that observed *in vitro* for both mutants (**Figure 1**) – and that might be due to alterations in OM permeability to nutrients and/or toxic compounds. On the other side, both mutants showed an opposed internalization pattern in HeLa non-professional phagocytes (**Figures 6E,F**) that does not correlate with their similar attenuation in mice. Since, in addition, other single and multiple mutants with analogous profiles in HeLa cells were not attenuated in mice (e.g., *B. ovis* Δ*omp10*Δ*omp31* and Δ*omp25*Δ*omp25c*) (**Table 1** and **Figure 6**), an altered behavior in HeLa cells of a *B. ovis* mutant does not imply attenuation in the mouse model.

The Δ*omp10*Δ*ugpB*Δ*omp31* mutant behaved in macrophages similarly to the Δ*omp31*Δ*cgs* and Δ*omp10*Δ*omp31*Δ*omp25c* mutants (**Figure 6B**). However, although during the first 3 W p.i. the three mutants showed a similar pattern of spleen colonization in mice (**Figure 2**), the Δ*omp10*Δ*ugpB*Δ*omp31* mutant was progressively cleared from spleen thereafter (**Figure 2D**). Therefore, additional defects, preventing the establishment of a chronic infection, must be present in the Δ*omp10*Δ*ugpB*Δ*omp31* mutant. It must be noted that after W5 p.i., concomitantly with the splenic dampening of CFU, the antibody response induced by *B. ovis* PA Δ*omp10*Δ*ugpB*Δ*omp31* was higher than that observed in mice infected with the parental strain (**Figure 2F**) (*P* < 0.05), despite the higher spleen colonization of the latter (**Figure 2A**). This increased humoral immunity might contribute to the clearance of the mutant but, at the same time, be a relevant characteristic for an attenuated vaccine. Clearing of the mutant after W3 p.i. could also be related with defects impairing its colonization and/or survival inside reservoir cells involved in sustaining the chronic phase of infection. In the case of *B. abortus*, alternatively activated macrophages – more abundant during the chronic phase of infection – have been described as preferential target cells for survival and replication in mice (Xavier et al., 2013). This preference has been related with an increase in glucose availability in these cells (Xavier et al., 2013), which adds new evidence about the relevant role of metabolism in the acute phase of infection and/or in sustaining persistent *Brucella* infection (Hong et al., 2000; Ronneau et al., 2016; Barbier et al., 2018). Since transport through membranes of metabolic substrates, metals, and other compounds is a key mechanism for bacterial homeostasis, the changes in the bacterial surface suffered by the Δ*omp10*Δ*ugpB*Δ*omp31* mutant might lead to an altered transport of essential molecules that could impair survival in the reservoir cells. However, these changes would not have dramatic effects under *in vitro* growth conditions, at least in the culture medium used in this work (**Figure 1**).

According to its attenuation profile, the Δ*omp10*Δ*ugpB* Δ*omp31* mutant was considered the best candidate to evaluate its vaccine properties against in the mouse model. Three weeks after the challenge, when *B. ovis* PA reaches its peak of infection in unvaccinated mice (**Figure 2**), neither the virulent strain nor the vaccine strain were detected in spleen, while both strains were detected – although in low levels – in some mice vaccinated with *B. melitensis* Rev1 (**Figure 8F**). The protective activity of *B. ovis* Δ*omp10*Δ*ugpB*Δ*omp31* was accompanied by a good antibody response against *B. ovis* PA whole cells that was significantly higher than that obtained with *B. melitensis* Rev1 by the time of challenge (**Figure 8F**) and even higher (*P* < 0.05) than that observed in mice infected with the parental strain (**Figure 2F**). The development of specific antibodies is critical for the protective activity against *B. melitensis* of both killed and live strains (Vitry et al., 2014) and was described as even more relevant than cell-mediated immunity in protecting against *B. ovis* infection (Jiménez de Bagüés et al., 1994). Accordingly, the strong humoral immune response elicited by *B. ovis* Δ*omp10*Δ*ugpB*Δ*omp31* could account for its remarkable protective activity against *B. ovis*. The similar levels of specific IgG_1_, IgG_2a_, and IgG_2b_ suggest a mixed Th1/Th2 response, also observed with other *B. ovis* PA attenuated mutants (Sancho et al., 2014), that is considered as an advantage for attenuated live vaccines in controlling both early and late events in *Brucella* infections (Vitry et al., 2014). *B. ovis* Δ*omp10*Δ*ugpB*Δ*omp31* also induced a memory immune response evidenced by the profile of cytokine secretion obtained with splenocytes of vaccinated mice after re-stimulation with *B. ovis* PA whole cells (**Figure 8**). The cytokine response, at least regarding the parameters evaluated in this work, resembled that obtained with the classical vaccine *B. melitensis* Rev1, the best available vaccine against *B. ovis* with proved protective activity in the natural host (OIE, 2017b). Both vaccination groups showed remarkable levels of IFN-g, key cytokine for the control of *Brucella* infections (Zhan and Cheers, 1993; Murphy et al., 2001; **Figure 8**). Important secretion of IL-10, described as an anti-inflammatory cytokine that also suppresses phagolysosome fusion in macrophages contributing to *Brucella* survival in the host (Corsetti et al., 2013; Hop et al., 2018), was also detected in both vaccination groups (**Figure 8**). However, since both vaccines provided good protection against infection, this increase in IL-10 levels could reflect a strong activation of the immune response after a second exposure, that would neutralize the pathogen but that could also have detrimental effects for the host and would need to be controlled.

Although mutants should be evaluated simultaneously to establish accurate comparisons, and other experimental conditions in the murine model would provide a more accurate information (e.g., different challenge doses, interval vaccination-challenge), the level of protection conferred by the Δ*omp10*Δ*ugpB*Δ*omp31* mutant against *B. ovis* infection seems better than that previously described for other *B. ovis* attenuated vaccines inoculated by the i.p. route (Sancho et al., 2014; Soler-Lloréns et al., 2014; Silva et al., 2015b). Additionally, a Δ*abcAB* mutant of *B. ovis*, which is defective in a species-specific ABC transporter (Silva et al., 2011) and that even encapsulated did not provide good protection level in mice (Silva et al., 2015b), protected against *B. ovis* in rams (Silva et al., 2015a). Accordingly, the results obtained in this work encourage the evaluation of *B. ovis* Δ*omp10*Δ*ugpB*Δ*omp31* as vaccine in the natural host. Moreover, the usefulness of *B. ovis* Δ*omp31*Δ*cgs* and Δ*omp10*Δ*omp31*Δ*omp25c* in rams should not be discarded, since the mouse model – although considered a good approach to evaluate the virulence of *Brucella* strains – has limitations (Kahl-McDonagh and Ficht, 2006; Grilló et al., 2012) and some virulent mutants in the mouse model were found attenuated in the natural host (Bellaire et al., 2003). Additionally, considering that they are avirulent in mice at low doses of infection, that they do not reach the maximum levels of infection at higher infection doses (**Figure 2**) and that they barely induce inflammation in spleen (**Figure 2**), the Δ*omp31*Δ*cgs* and Δ*omp10*Δ*omp31*Δ*omp25c* mutants might be unable to produce epididymitis in rams. In addition to the protective properties, the three attenuated mutants would provide diagnostic advantages. They would not interfere with the serological diagnosis of infections caused by smooth *Brucella* and, since they are defective in major Omp31 that has been proposed as an interesting diagnostic antigen for *B. ovis* infection (Vizcaíno et al., 2001b), they would favor the differentiation between vaccinated and infected animals by using Omp31 as diagnostic antigen.

In addition to the development of a vaccine candidate against *B. ovis*, the results obtained in this work provide new information about the relationships among cell-envelope molecules of *B. ovis* and the peculiar characteristics of its OM. This knowledge could help to explain why *B. ovis*, despite its reported genome degradation (Tsolis et al., 2009), establishes persistent infections in its natural host whereas rough mutants derived from S *Brucella* do not or why its pathogenicity differs from that of *B. melitensis* in the same preferred host.

## Author Contributions

NV, RS-M, and PS conceived the study and wrote the paper. RS-M, PS, NV, MM, CT, AC, and MZ performed the experimental work. All authors participated in the presentation and discussion of the results and in the revision of the manuscript.

## Conflict of Interest Statement

The authors declare that the research was conducted in the absence of any commercial or financial relationships that could be construed as a potential conflict of interest.
